# Inhibition of Prostate Smooth Muscle Contraction by Inhibitors of Polo-Like Kinases

**DOI:** 10.3389/fphys.2018.00734

**Published:** 2018-06-15

**Authors:** Martin Hennenberg, Paul Kuppermann, Qingfeng Yu, Annika Herlemann, Alexander Tamalunas, Yiming Wang, Beata Rutz, Anna Ciotkowska, Frank Strittmatter, Christian G. Stief, Christian Gratzke

**Affiliations:** Department of Urology, Ludwig Maximilian University of Munich, Munich, Germany

**Keywords:** benign prostatic hyperplasia (BPH), lower urinary tract symptoms (LUTS), prostate smooth muscle contraction, α_1_-adrenoceptor, α_1_-blocker, polo-like kinase (PLK)

## Abstract

**Background:** Prostate smooth muscle contraction plays an important role for pathophysiology and treatment of male lower urinary tract symptoms (LUTS) but is incompletely understood. Because the efficacy of available medication is limited, novel options and improved understanding of prostate smooth muscle contraction are of high demand. Recently, a possible role of polo-like kinase 1 (PLK1) has been suggested for smooth muscle contraction outside the lower urinary tract. Here, we examined effects of PLK inhibitors on contraction of human prostate tissue.

**Methods:** Prostate tissues were obtained from radical prostatectomy. RT-PCR, Western blot and immunofluorescence were performed to detect PLK expression and phosphorylated PLK. Smooth muscle contractions were induced by electric field stimulation (EFS), α_1_-agonists, endothelin-1, or the thromboxane A_2_ analog U46619 in organ bath.

**Results:** RT-PCR, Western blot, and immunofluorescence suggested expression of PLK1 in the human prostate, which may be located and active in smooth muscle cells. EFS-induced contractions of prostate strips were reduced by SBE 13 (1 μM), cyclapolin 9 (3 μM), TAK 960 (100 nM), and Ro 3280 (100 nM). SBE 13 and cyclapolin 9 inhibited contractions by the α_1_-agonists methoxamine, phenylephrine, and noradrenaline. In contrast, no effects of SBE 13 or cyclapolin 9 on endothelin-1- or U46619-induced contractions were observed.

**Conclusion:** Alpha1-adrenergic smooth muscle contraction in the human prostate can be inhibited by PLK inhibitors. PLK-dependent signaling may be a new pathway, which promotes α_1_-adrenergic contraction of prostate smooth muscle cells. As contractions by endothelin and U46619 are not susceptible to PLK inhibition, this reflects divergent regulation of adrenergic and non-adrenergic prostate smooth muscle contraction.

## Introduction

Male lower urinary tract symptoms (LUTS) suggestive of benign prostatic hyperplasia (BPH) are commonly caused by bladder outlet obstruction (BOO), which is driven by increased prostate smooth muscle tone and prostate growth (Caine et al., 1976; Hennenberg et al., 2014). Induction of prostate smooth muscle relaxation is an important strategy for medical treatment of voiding symptoms (Caine et al., 1976; Oelke et al., 2013; Hennenberg et al., 2014). The most important option are α_1_-adrenoceptor antagonists, as they may reduce symptoms by inhibition of α_1_-adrenergic prostate smooth muscle contraction and subsequent improvement of urethral obstruction and bladder emptying (Caine et al., 1976; Oelke et al., 2013; Hennenberg et al., 2014). However, their efficacy is limited, so that novel options and better understanding of the regulation of prostate smooth muscle contraction are required (Oelke et al., 2013; Hennenberg et al., 2014, 2017).

For decades, it has been assumed that prostate smooth muscle contraction is promoted by three intracellular signaling pathways, including phospholipase C/IP_3_, diacylglycerol/protein kinase C, and RhoA/Rho kinase, which are activated by α_1_-adrenoceptors and by receptors for endothelin-1 and thromboxane A_2_ (Christ and Andersson, 2007; Hennenberg et al., 2014). This concept has been recently challenged, when it became increasingly obvious, that much more signaling pathways may be involved in promotion of prostate smooth muscle contraction (Hennenberg et al., 2014). These may include several GTPases and kinases, as smooth muscle contraction of the human prostate was inhibited by inhibitors for RacGTPases, focal adhesion kinases, p21-activated kinases, and src family kinases (Kunit et al., 2014; Wang et al., 2015, 2016a,b). Together, this suggested that mechanisms regulating or promoting prostate smooth muscle contraction may be much more complex than previously assumed.

Polo-like kinases (PLKs) are a group of serine-threonine kinases, which have mostly been associated with regulation of cell cycle and promotion of proliferation. In addition to this function, recent studies suggested a role of PLK1 for contraction of airway and vascular smooth muscle contraction (Li et al., 2016; de Carcer et al., 2017). Moreover, several small molecule inhibitors for PLK1 have now become commercially available. Therefore, we here studied the effects of PLK1 inhibitors on contraction of human prostate tissue and possible expression of PLK1 in the human prostate.

## Materials and Methods

### Human Prostate Tissues

**FIGURE 1:**
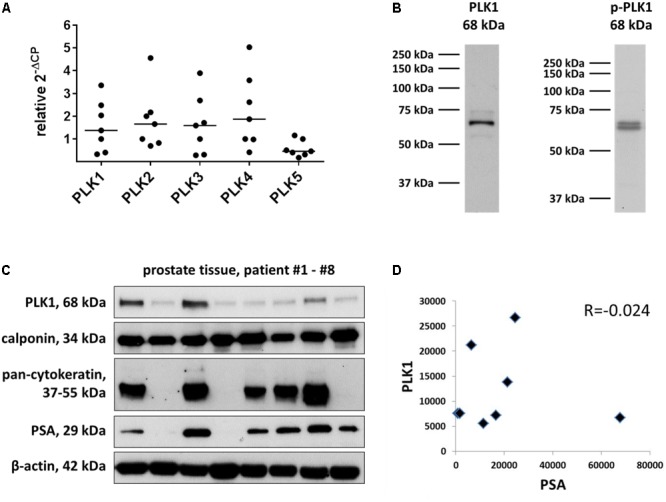
Detection of PLK in human prostate tissue. Analyses were performed by RT-PCR to detect mRNA of different PLK isoforms **(A)**, or by Western blots to detect putative PLK1 protein **(B,C)**. Data in **(A)** are ΔΔCP values [2ˆ-(Ct_target_-Ct_GAPDH_), normalized to each other] and median values (bar), from prostate tissues from *n* = 7 patients. In **(B)**, bands from all included samples are shown, with sizes matching the expected and indicated molecular weights of proteins. Western blot analysis included calponin as a marker for smooth muscle cells, pan-cytokeratin as a marker of endothelial cells (glands), and prostate-specific antigen (PSA) as a marker for benign prostatic hyperplasia. In **(C)**, values (arbitrary units) after densitometric quantification of Western blots were plotted in diagrams, and subjected to Spearman’s correlation analysis. In **(D)**, correlation analysis for band intensities of PLK1 and PSA are shown.

Human prostate tissues were obtained from patients (*n* = 157) undergoing radical prostatectomy for prostate cancer. Patients who underwent previous transurethral resection of the prostate (TURP) were excluded. This study was carried out in accordance with the Declaration of Helsinki of the World Medical Association, and has been approved by the ethics committee of the Ludwig Maximilian University of Munich, Munich, Germany. Informed consent was obtained from all patients. Samples and data were collected and analyzed anonymously. Samples were taken immediately after prostatectomy, following macroscopical examination by an uro-pathologist. All tissues were taken from the periurethral zone, considering that most prostate cancers arise in the peripheral zone (Pradidarcheep et al., 2011; Shaikhibrahim et al., 2012). Upon pathologic evaluation, only tissue samples which did not exhibit histological signs of neoplasia, cancer, or inflammation were collected. BPH is present in 80–83% of patients with prostate cancer (Alcaraz et al., 2009; Orsted and Bojesen, 2013). The content of prostate-specific antigen (PSA) increases with the degree of BPH, so that varying PSA content (**Figure 1**) reflects divergent degree of BPH in prostate samples from different patients (Levitt and Slawin, 2007). For macroscopic examination and sampling, the prostate was opened by a single longitudinal cut from the capsule to the urethra. Subsequently, both intersections were checked macroscopically for any obvious tumor infiltration. Because tumors are usually located to the peripheral zone, tumor infiltration in the periurethral zone (where sampling was performed) was very rare (found in less than 1% of prostates). Prostates showing tumors in the periurethral zone on macroscopic inspection were not subjected to sampling and were not included in this study. Organ bath studies were performed immediately after sampling, while samples for molecular analyses were shock frozen in liquid nitrogen and stored at -80°C.

### Real Time Polymerase Chain Reaction (RT-PCR)

RNA from frozen prostate tissues or cells was isolated using the RNeasy Mini kit (Qiagen, Hilden, Germany). For isolation from tissues, 30 mg of tissue were homogenized using the FastPrep^®^-24 system with matrix A (MP Biomedicals, Illkirch, France). RNA concentrations were measured spectrophotometrically. Reverse transcription to cDNA was performed with 1 μg of isolated RNA using the Reverse Transcription System (Promega, Madison, WI, United States). RT-PCR for PLK isoforms 1–5 and glyceraldehyde 3-phosphate dehydrogenase (GAPDH) was performed with a Roche Light Cycler (Roche, Basel, Switzerland) using primers provided by Qiagen (Hilden, Germany) as ready-to-use mixes, based on the RefSeq accession numbers NM_005030 for PLK1, NM_001252226 for PLK2, NM_004073 for PLK3, NM_001190799 for PLK4, NM_001243079 for PLK5, and NM_002046 for GAPDH. PCR reactions were performed in a volume of 25 μl containing 5 μl LightCycler^®^ FastStart DNA MasterPlus SYBR Green I (Roche, Basel, Switzerland), 1 μl template, 1 μl primer, and 18 μl water. Denaturation was performed for 10 min at 95°C, and amplification with 45 cycles of 15 s at 95°C followed by 60 s at 60°C. The specificity of primers and amplification was demonstrated by subsequent analysis of melting points, which revealed single peaks for each target. Results were expressed using the ΔΔCP method, where number of cycles (Ct) at which the fluorescence signal exceeded a defined threshold for GAPDH was subtracted from Ct values for PLK (Ct_PLK_-Ct_GAPDH_ = ΔCP), and values were calculated as 2ˆ- ΔCP and normalized to each other.

### Western Blot Analysis

Frozen prostate tissues were homogenized in a buffer containing 25 mM Tris/HCl, 10 μM phenylmethanesulfonyl fluoride, 1 mM benzamidine, and 10 μg/ml leupeptine hemisulfate, using the FastPrep^®^-24 system with matrix A (MP Biomedicals, Illkirch, France). After centrifugation (20,000 *g*, 4 min), supernatants were assayed for protein concentration using the Dc-Assay kit (Biorad, Munich, Germany) and boiled for 10 min with sodium dodecyl sulfate (SDS) sample buffer (Roth, Karlsruhe, Germany). Samples (20 μg/lane) subjected to SDS-polyacrylamide gel electrophoresis, and proteins were blotted on Protran nitrocellulose^®^ membranes (Schleicher & Schuell, Dassel, Germany). Membranes were blocked with phosphate-buffered saline (PBS) containing 5% milk powder (Roth, Karlsruhe, Germany) over night, and incubated with rabbit anti PLK1 (208G4) (#4513), rabbit anti phospho-PLK1 (threonine 210) (#5472), mouse monoclonal anti pan-cytokeratin(sc-8018) (Santa Cruz Biotechnology, Santa Cruz, CA, United States), mouse monoclonal anti calponin 1/2/3 (sc-136987) (Santa Cruz Biotechnology, Santa Cruz, CA, United States), mouse monoclonal anti PSA (sc-7316) (Santa Cruz Biotechnology, Santa Cruz, CA, United States), rabbit anti phospho-vimentin (serine 56) (#3877), mouse anti vimentin (#3390), mouse anti phospho-myosin light chain (MLC) 2 (serine 19) (#3675), rabbit anti myosin light chan 2 (#8505), or mouse monoclonal anti β-actin antibody (sc-47778) (Santa Cruz Biotechnology, Santa Cruz, CA, United States) (if not other stated, from Cell Signaling Technology, Danvers, MA, United States).

Primary antibodies were diluted in PBS containing 0.1% Tween 20 (PBS-T) and 5% milk powder. Subsequently, detection was continued using secondary biotinylated horse anti mouse or horse anti goat IgG (BA-1000, BA-2000, BA-9500) (Vector Laboratories, Burlingame, CA, United States), followed by incubation with avidin and biotinylated horseradish peroxidase (HRP) from the “Vectastain ABC kit” (Vector Laboratories, Burlingame, CA, United States) both diluted 1:200 in PBS. Membranes were washed with PBS-T after any incubation with primary or secondary antibodies, or biotin-HRP. Finally, blots were developed with enhanced chemiluminescence (ECL) using ECL Hyperfilm (GE Healthcare, Freiburg, Germany). Intensities of resulting bands for PLK1 and PSA were quantified densitometrically using “Image J” (National Institutes of Health, Bethesda, MD, United States), and values (arbitrary units) were plotted against each other and subjected to Spearman’s correlation analysis.

### Immunofluorescence

Human prostate specimens, embedded in optimal cutting temperature (OCT) compound, were snap-frozen in liquid nitrogen and kept at -80°C. Sections (8 μm) were cut in a cryostat and collected on Superfrost^®^ microscope slides. Sections were post-fixed in methanol at -20°C and blocked in 1% bovine serum albumin before incubation with primary antibody over night at room temperature. For double labeling, the following primary antibodies were used: rabbit anti PLK1 (208G4) (#4513) (Cell Signaling Technology, Danvers, MA, United States), rabbit anti phospho-PLK1 (threonine 210) (#5472) (Cell Signaling Technology, Danvers, MA, United States), mouse anti pan-cytokeratin (sc-8018), or mouse anti calponin 1/2/3 (sc-136987) (if not other stated, from Santa Cruz Biotechnology, Santa Cruz, CA, United States). Binding sites were visualized using Cy3-conjugated goat anti mouse IgG (AP124C), fluorescein isothiocyanate- (FITC-) conjugated rabbit anti goat IgG (AP106F) (both from Millipore, Billerica, MA, United States, and Cy5-conjugated goat anti rabbit IgG (ab6564) (Abcam, Cambridge, United Kingdom). Nuclei were counterstained with 4′,6′-diamidino-2-phenylindole-dihydrochloride (DAPI) (Invitrogen, Camarillo, CA, United States). Immunolabeled sections were analyzed using a laser scanning microscope (Leica SP2, Wetzlar, Germany). Fluorescence was recorded with separate detectors. Control stainings without primary antibodies did not yield any signals.

### Tension Measurements

Prostate strips (6 × 3 × 3 mm) were mounted in 10 ml aerated (95% O_2_ and 5% CO_2_) tissue baths (Danish Myotechnology, Aarhus, Denmark), containing Krebs–Henseleit solution (37°C, pH 7.4) with following composition: 118 mM NaCl, 4.7 mM KCl, 2.55 mM CaCl_2_, 1.2 mM KH_2_PO_4_, 1.2 mM MgSO_4_, 25 mM NaHCO_3_, 7.5 mM glucose. In each single experiment, four strips were obtained from the same prostate, and allocated to both the control (without inhibitor) and inhibitor group (two strips per group, resulting in duplicate determination for each group in each single experiment). Consequently, control and inhibitor curves in each diagram were obtained from the same prostates, but different prostates were examined for different diagrams. Therefore, and considering that prostate tissues from radical prostatectomy may show considerable heterogeneity (as shown in the results section), statistical comparisons were only performed between groups within the same series (i.e., containing tissues from the same prostates for inhibitor and control group), but not between series obtained from different prostates (i.e., not across different series of organ bath experiments). The amount of solvent differed between series, due to divergent inhibitor concentrations (100 nM, 1 μM, 3 μM). Again, this precludes any comparison between contraction levels in different series. Only one curve was recorded with each sample (agonist or EFS, either with DMSO or inhibitor).

After mounting in organ bath chambers, preparations were stretched to 4.9 mN and left to equilibrate for 45 min. In the initial phase of the equilibration period, spontaneous decreases in tone are usually observed. Therefore, tension was adjusted three times during the equilibration period, until a stable resting tone of 4.9 mN was attained. After the equilibration period, maximum contraction induced by 80 mM KCl was assessed. Subsequently, chambers were washed three times with Krebs–Henseleit solution for a total of 30 min. Cumulative concentration response curves for noradrenaline, phenylephrine, methoxamine, endothelin-1, and for U46619, or frequency response curves induced by electric field stimulation (EFS) were created 30 min after addition of SBE 13 (1 μM), cyclapolin 9 (3 μM), or dimethylsulfoxide (DMSO) for controls. Application of EFS simulates action potentials, resulting in the release of endogenous neurotransmitters, including norepinephrine. Using the inhibitor for neurotransmitter release, tetrodotoxin, it has been previously demonstrated, that this accounts for two-thirds of EFS-induced contraction in the human prostate (Angulo et al., 2012). For EFS, tissue strips were placed between two parallel platinum electrodes connected to a Grass S48 stimulator (Danish Myotechnology, Denmark). Square pulses with durations of 1 ms were applied with a voltage of 50 V, for a train duration of 10 s and using a delay of 1 ms between single pulses. EFS-induced contractile responses were studied at frequencies of 2, 4, 8, 16, and 32 Hz, with train intervals of 30 s between stimulations.

For calculation of agonist- or EFS-induced contractions, tensions (peak height in EFS-induced contractions and maximum contractions following agonist-exposure) were expressed as % of KCl-induced contractions, as this may correct different stromal/epithelial ratios, different smooth muscle content, varying degree of BPH, or any other heterogeneity between prostate samples and patients. As KCl-induced contractions were assessed before application of PLK inhibitors, effects of inhibitors can be seen despite diverging expression levels of PLK1.

### Phosphorylation Studies

Tissues from each included prostate were cut into several small strips (6 × 1 × 1 mm), which were then allocated to two or three samples (control group and agonist group, or one control group and two inhibitor groups). Consequently, all series had identical group sizes, and in each single experiment, tissue from the same patient was used for all groups. Incubation of samples with inhibitors, agonists and solvent (controls) was performed in 6-well plates filled with custodiol solution. After an equilibration period of 20 min, inhibitors, agonists and solvent were added, and plates were kept at 37°C under continuous shaking for indicated periods. Therefore, samples of the control groups were kept under experimental conditions for the same periods as their corresponding agonist or inhibitor groups. Following incubations, tissues were shock frozen with liquid nitrogen, and subjected to Western blot analysis for phospho-PLK, PLK, phospho-vimentin, vimentin, phospho-MLC, MLC, or β-actin. Each setting was repeated in several independent experiments using different prostates, to obtain groups sizes as indicated. Intensities of resulting bands were quantified densitometrically using “Image J” (National Institutes of Health, Bethesda, MD, United States). For semiquantitative calculation, values of each sample were normalized to the mean of the corresponding control group, so that agonist and inhibitor groups are expressed as percent (%) of the corresponding control group.

### Drugs and Nomenclature

*N*-[[4-[(6-Chloro-3-pyridinyl)methoxy]-3-methoxyphenyl]methyl]-3,4-dimethoxybenzeneethanamine hydrochloride (SBE 13), 7-Nitro-5-(trifluoromethyl)-2-benzothiazolecarboxamide-3-oxide (cyclapolin 9), 4-[(9-Cyclopentyl-7,7-difluoro-6,7,8,9-tetrahydro-5-methyl-6-oxo-5H-pyrimido[4,5-b][1,4]diazepin-2-yl) amino]-2-fluoro-5-methoxy-*N*-(1-methyl-4-piperidinyl)benzamide hydrochloride (TAK 960), and 4-[(9-Cyclopentyl-7,7-difluoro-6,7,8,9-tetrahydro-5-methyl-6-oxo-5H-pyrimido[4,5-b] [1,4]diazepin-2-yl)amino]-3-methoxy-*N*-(1-methyl-4-piperidinyl) benzamide (Ro 3280) are selective PLK1 inhibitors. Stock solutions (10 mM) were prepared in DMSO, and stored at -20°C until use. Phenylephrine ((R)-3-[-1-hydroxy-2-(methylamino)ethyl]phenol) and methoxamine (α-(1-Aminoethyl)-2,5-dimethoxybenzyl alcohol) are selective agonists for α_1_-adrenoceptors. U46619 ((Z)-7-[(1S,4R,5R,6S)-5-[(E,3S)-3-hydroxyoct-1-enyl]-3-oxabicyclo[2.2.1]heptan-6-yl]hept-5- enoic acid) is an analog of thromboxane A_2_ (TXA_2_) and frequently used as an agonist for thromboxane A_2_ receptors. Aqueous stock solutions of phenylephrine and noradrenaline (10 mM) were freshly prepared before each experiment. Stock solutions of U46619 were prepared in ethanol, and stored at -80°C until use. Aqueous stock solutions of endothelin-1 were stored at -20°C until used. SBE 13, cyclapolin 9, TAK 960, Ro 3280, and U46619 were obtained from Tocris (Bristol, United Kingdom), phenylephrine, methoxamine, and noradrenaline were obtained from Sigma (Munich, Germany), and endothelin-1 from Enzo Life Sciences (Lörrach, Germany).

### Statistical Analysis

Data are presented as means ± standard error of the mean (SEM) with the indicated number (n) of experiments. One-way analysis of variance (ANOVA) and multivariate ANOVA were used for paired or unpaired observations. *P*-values < 0.05 were considered statistically significant.

## Results

### Detection of PLK1 in Human Prostate Tissues

By RT-PCR, mRNAs for all five PLK isoforms were detectable in human prostate tissues (**Figure 1A**). The content of mRNA for PLK isoforms 1-4 varied considerably between different prostates (**Figure 1A**). As a role in smooth muscle contraction may only be supposed for PLK1, Western blot analysis was performed for PLK1. Detection with an antibody raised against PLK1 revealed bands with sizes matching the expected molecular weight of PLK1, while other bands were almost completely absent (**Figure 1B**). These bands were observed in each prostate sample included in this analysis, despite obvious variations in intensity between bands obtained with samples from different patients (**Figure 1C**). Following semiquantitative evaluation of bands, correlation analysis was performed for PLK1 and PSA. No correlation was observed between intensities of assumed PLK1 and PSA bands (*R* = -0.024) (**Figure 1D**).

**FIGURE 2:**
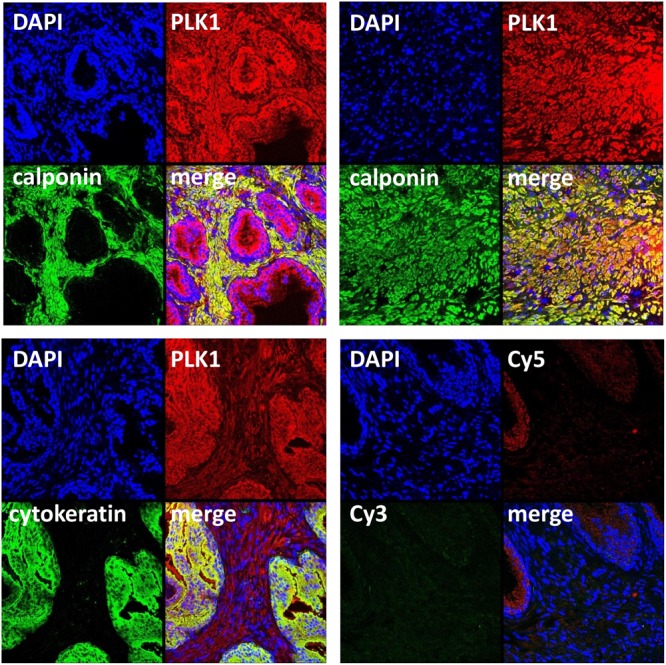
Immunofluorescence staining for PLK1 of human prostate tissues. Sections were double labeled with antibodies for PLK1, calponin (marker for smooth muscle cells) or pan-cytokeratin (marker for glandular epithelial cells). Yellow color in merged pictures indicates colocalization of targets. Shown are representative stainings from series with tissues from *n* = 5 patients for each combination, performed with tissues showing typical architecture composed of glands and stroma, and a tissue from an extended stromal area showing only calponin-positive smooth muscle cells but no glands **(upper panel, right image)**. Negative controls were performed without primary antibodies but Cy3- and Cy5-coupled secondary antibodies **(lower panel, right image)**.

Immunofluorescence stainings were performed using antibodies raised against PLK1 and phospho-PLK1 (threonine 210). Similar to the antibody raised against PLK1, the antibody raised against phospho-PLK1 revealed bands with sizes matching the expected molecular weight of PLK1, while other bands were lacking (**Figure 1B**). Most samples of prostate tissue showed the typical architecture, composed of stroma with calponin-positive smooth muscle cells, and glands with pan-cytokeratin-positive epithelial cells (**Figure 2**). In addition to the typical architecture, some parts were characterized by large areas without glands and contained only stroma and predominantly calponin-positive cells (**Figure 2**). Immunoreactivity for the PLK1 antibody was observed in the stroma and in glands (**Figure 2**). In the stroma, PLK1 immunoreactivity was strong and colocalized with calponin in both types of prostate tissue, suggesting localization of this immunoreactivity in smooth muscle cells of areas with normal architecture, and in areas with extended stroma (**Figure 2**). In glands, immunoreactivity for PLK1 colocalized with pan-cytokeratin, suggesting localization in the glandular epithelium (**Figure 2**).

**FIGURE 3:**
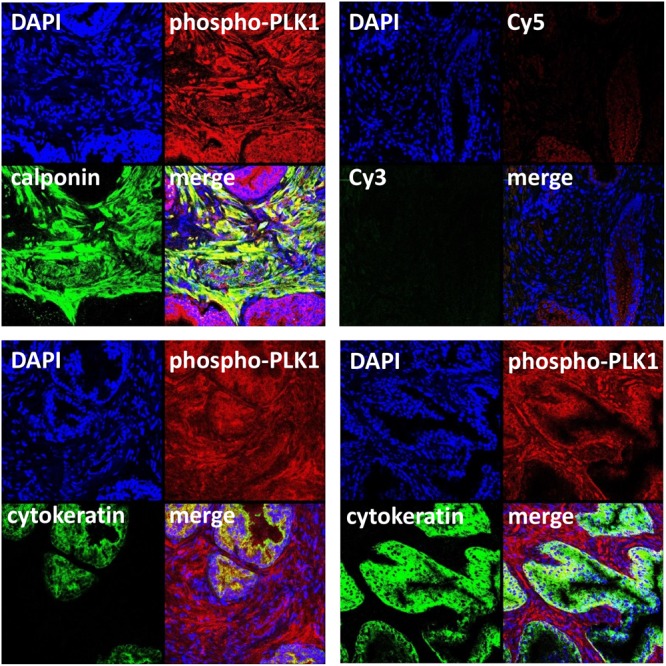
Immunofluorescence staining for phospho-PLK1 of human prostate tissues. Sections were double labeled with antibodies for phospho-PLK1 (threonine 210), calponin (marker for smooth muscle cells) or pan-cytokeratin (marker for glandular epithelial cells). Yellow color in merged pictures indicates colocalization of targets. Shown are representative stainings from series with tissues from *n* = 5 patients for each combination, and examples with and without glandular colocalization of phospho-PLK1 with pan-cytokeratin **(lower panels)**. Negative controls were performed without primary antibodies but Cy3- and Cy5-coupled secondary antibodies **(upper panel, right image)**.

Immunoreactivity for the phospho-PLK1 antibody was observed in the stroma, where it was ubiquitous and strong, and to a limited degree in glands (**Figure 3**). There was immunoreactivity for phospho-PLK1 colocalized with calponin in the stroma, suggesting the presence of active PLK1 in smooth muscle cells (**Figure 3**). Colocalization of phospho-PLK1 immunoreactivity with pan-cytokeratin was observed in some, but not all glands (**Figure 3**).

### Effects of SBE 13, Cyclapolin 9, TAK 960, and Ro 3280 on EFS-Induced Contractions

**FIGURE 4:**
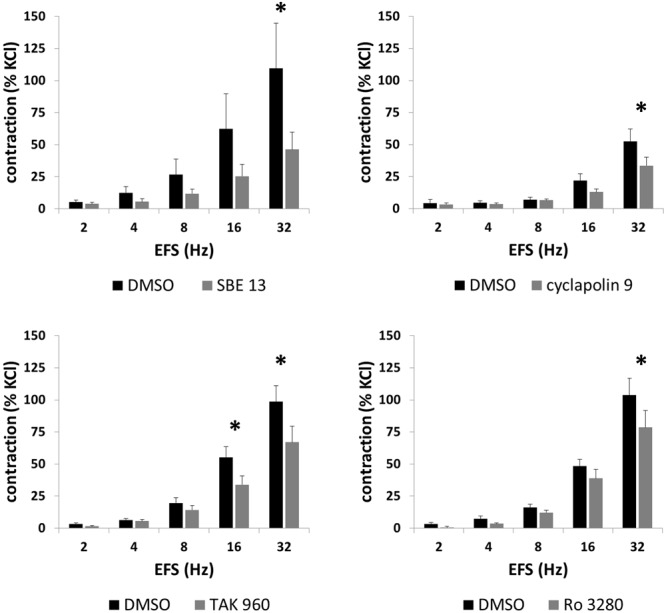
Effects of SBE 13 (1 μM), cyclapolin 9 (3 μM), TAK 960 (100 nM), and Ro 3280 (100 nM) on EFS-induced prostate contractions. In an organ bath, contractions of human prostate strips were induced by EFS. Effects of inhibitors on contractions were compared with corresponding controls (DMSO) in separate sets of experiments. To eliminate heterogeneities including any individual variations, different degree of BPH, or varying smooth muscle content (compare **Figure 1**), tensions have been expressed as % of highmolar KCl-induced contraction, which was assessed before application of inhibitors or solvent. Data are means ± SEM from series with tissues from *n* = 7 patients/group for SBE 13, *n* = 7 patients/group for cyclapolin 9, *n* = 7 patients/groups for TAK 960, and *n* = 10 patients/group for Ro 3280. For each diagram, samples from each patient were allocated to both the control and inhibitor groups, so that both groups in each diagram are obtained from the same tissues (^∗^*p* < 0.05 for control vs. inhibitor).

EFS (2–32 Hz) induced frequency-dependent contractions of prostate strips, which were inhibited by SBE 13 (1 μM), cyclapolin 9 (3 μM), TAK 960 (100 nM), and Ro 3280 (100 nM) (**Figure 4**). Two-way ANOVA was conducted to compare inhibitor and control groups, and indicated that the inhibition by SBE 13 (*p* < 0.02), cyclapolin 9 (*p* < 0.03), TAK 960 (*p* < 0.007), and Ro 3280 (*p* < 0.04) was significant. Multivariate analysis revealed that inhibition was significant for all four inhibitors at least at 32 Hz (**Figure 4**).

### Effects of SBE 13 and Cyclapolin 9 on Methoxamine-Induced Contractions

**FIGURE 5:**
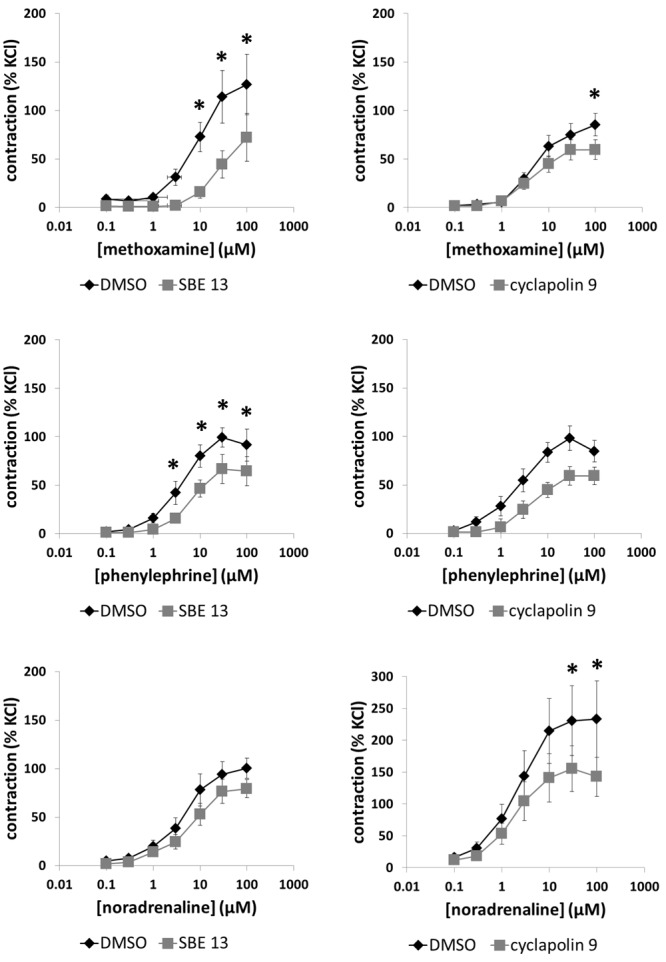
Effects of SBE 13 (1 μM) and cyclapolin 9 (3 μM) on α_1_-adrenoceptor-induced prostate contractions. In an organ bath, contractions of human prostate strips were induced by the α_1_-adrenoceptor agonists methoxamine, phenylephrine, or noradrenaline. Effects of inhibitors on contractions were compared with corresponding controls (DMSO) in separate sets of experiments. To eliminate heterogeneities including any individual variations, different degree of BPH, or varying smooth muscle content (compare **Figure 1**), tensions have been expressed as % of highmolar KCl-induced contraction, which was assessed before application of inhibitors or solvent. Data are means ± SEM from series with tissues from *n* = 10 patients/group for noradrenaline/SBE 13, *n* = 5 patients/group for noradrenaline/cyclapolin 9, *n* = 5 patients/group for methoxamine/SBE 13, *n* = 13 patients/group for methoxamine/cyclapolin 9, *n* = 8 patients/group for phenylephrine/SBE 13, and *n* = 9 patients/group for phenylephrine/cyclapolin 9. For each diagram, samples from each patient were allocated to both the control and inhibitor groups, so that both groups in each diagram are obtained from the same tissues (^∗^*p* < 0.05 for control vs. inhibitor).

Methoxamine (0.1–100 μM) induced concentration-dependent contractions of prostate strips, which were inhibited by SBE 13 (1 μM) and cyclapolin 9 (3 μM) (**Figure 5**). Two-way ANOVA was conducted to compare inhibitor and control groups, and indicated that the inhibition by SBE 13 (*p* < 0.002) and cyclapolin 9 (*p* < 0.02) was significant. Multivariate analysis revealed that inhibition by SBE 13 was significant at 10–100 μM of methoxamine, and by cyclapolin 9 at 100 μM of methoxamine (**Figure 5**).

### Effects of SBE 13 and Cyclapolin 9 on Phenylephrine-Induced Contractions

Phenylephrine (0.1–100 μM) induced concentration-dependent contractions of prostate strips, which were inhibited by SBE 13 (1 μM) and cyclapolin 9 (3 μM) (**Figure 5**). Two-way ANOVA was conducted to compare inhibitor and control groups, and indicated that the inhibition by SBE 13 (*p* < 0.002) was significant. Multivariate analysis revealed that inhibition by SBE 13 was significant at 3–100 μM of phenylephrine (**Figure 5**).

### Effects of SBE 13 and Cyclapolin 9 on Noradrenaline-Induced Contractions

Noradrenaline (0.1–100 μM) induced concentration-dependent contractions of prostate strips, which were inhibited by SBE 13 (1 μM) and cyclapolin 9 (3 μM) (**Figure 5**). Two-way ANOVA was conducted to compare inhibitor and control groups, and indicated that the inhibition by SBE 13 (*p* < 0.01) and cyclapolin 9 (*p* < 0.002) was significant. Multivariate analysis revealed that inhibition by cyclapolin 9 was significant at 30 and 100 μM of noradrenaline (**Figure 5**).

### Effects of SBE 13 and Cyclapolin 9 on Endothelin-1-Induced Contractions

**FIGURE 6:**
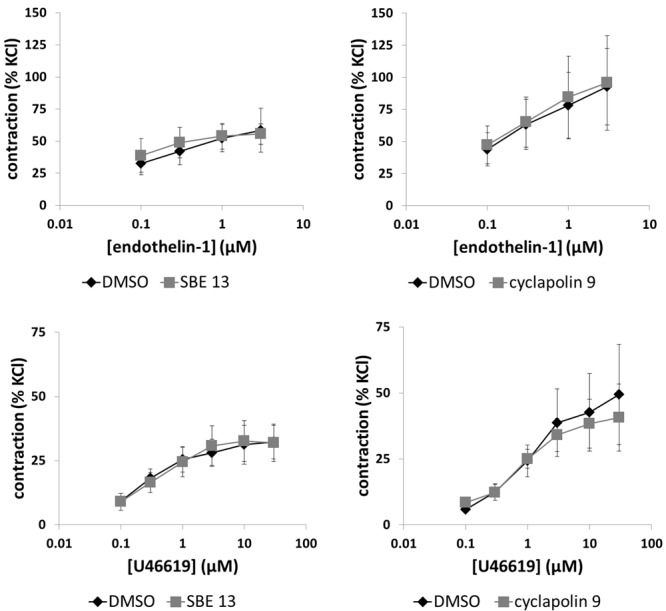
Effects of SBE 13 (1 μM) and cyclapolin 9 (3 μM) on non-adrenergic prostate contractions. In an organ bath, contractions of human prostate strips were induced by endothelin-1, or by the thromboxane A_2_ analog U46619. Effects of inhibitors on contractions were compared with corresponding controls (DMSO) in separate sets of experiments. To eliminate heterogeneities including any individual variations, different degree of BPH, or varying smooth muscle content (compare **Figure 1**), tensions have been expressed as % of highmolar KCl-induced contraction, which was assessed before application of inhibitors or solvent. Data are means ± SEM from series with tissues from *n* = 3 patients/group for endothelin-1/SBE 13, *n* = 3 patients/groups for endothelin-1/cyclapolin 9, *n* = 9 patients/group for U46619/SBE 13, and *n* = 6 patients/group for U46619/cyclapolin 9. For each diagram, samples from each patient were allocated to both the control and inhibitor groups, so that both groups in each diagram are obtained from the same tissues.

Endothelin-1 (0.1–3 μM) induced concentration-dependent contractions of prostate strips (**Figure 6**). Two series of experiments did not provide a basis to assume that SBE 13 (1 μM) or cyclapolin 9 (3 μM) may inhibit endothelin-1-induced contractions (**Figure 6**), so that these exploratory series were not continued after three independent experiments in each series.

### Effects of SBE 13 and Cyclapolin 9 on U46619-Induced Contractions

U46619 (0.1–30 μM) induced concentration-dependent contractions of prostate strips (**Figure 6**). SBE 13 (1 μM) and cyclapolin 9 (3 μM) did not alter U46619-induced contractions (**Figure 6**).

### Effects of Contractile Agonists on PLK Phosphorylation

**FIGURE 7:**
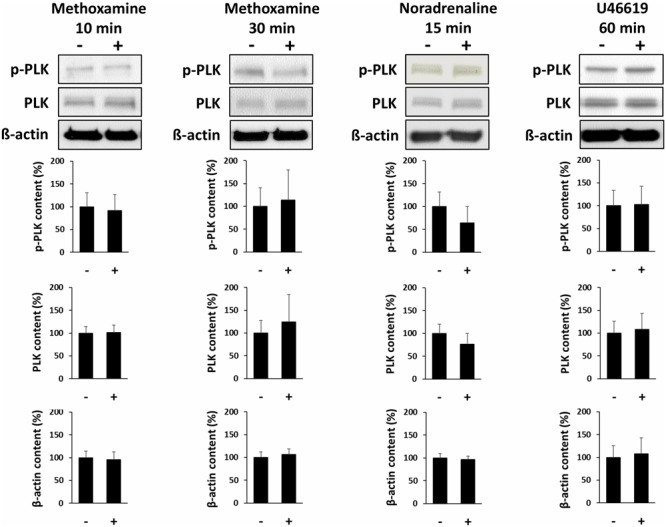
Effects of contractile agonists on PLK phosphorylation. Prostate tissues were stimulated with different agonists and for different periods in separate sets of experiments. Subsequently, PLK phosphorylation at threonine 210 was compared between stimulated samples and corresponding controls (exposed to identical experimental conditions) by Western blot analysis using a phospho-specific antibody. Stimulation was performed with the α_1_-adrenoceptor agonist methoxamine (30 μM) for 10 or 30 min, with noradrenaline (30 μM) for 15 min, or with the thromboxane A_2_ analog U46619 (30 μM) for 60 min. In each series, tissues in the agonist and corresponding control group were obtained from the same prostates. Shown are representative Western blots and quantification of all experiments, from series with *n* = 4 patients for methoxamine 10 min, *n* = 4 patients for methoxamine 30 min, *n* = 5 patients for noradrenaline 15 min, and *n* = 6 patients for noradrenaline 60 min.

Incubation of prostate tissues with methoxamine (30 μM) for 10 or 30 min, with noradrenaline (30 μM) for 30 min, or with U46619 (30 μM) for 60 min did not change the average content of phospho-PLK (threonine 210) or total PLK (**Figure 7**).

### Effects of PLK Inhibitors on Phosphorylation of Vimentin and MLC

**FIGURE 8:**
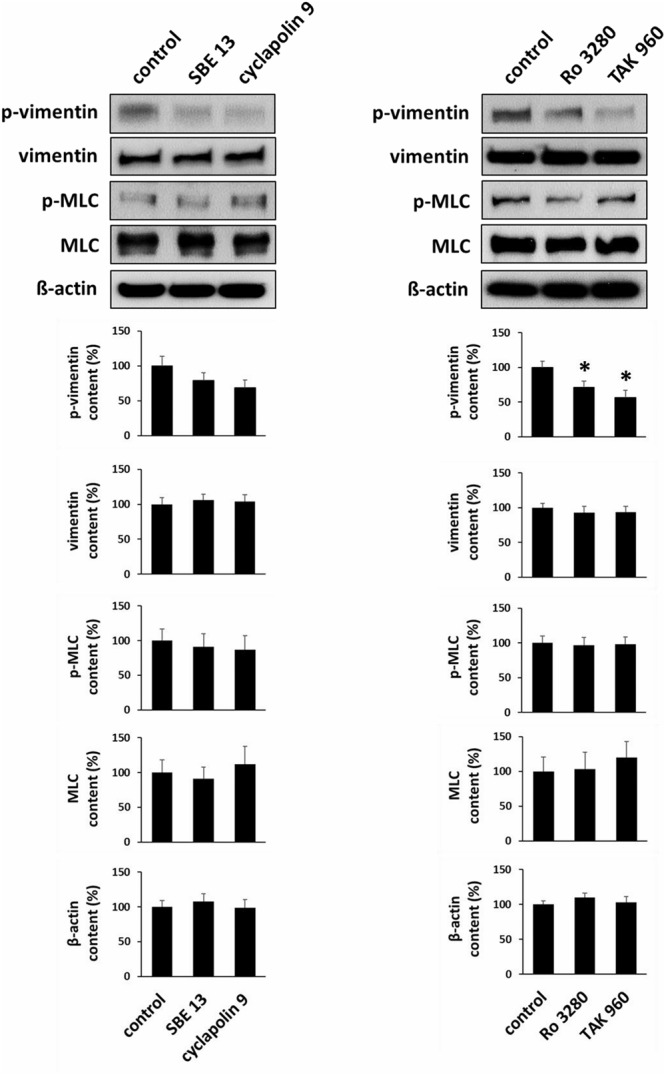
Effects of PLK inhibitors on phosphorylation of vimentin and MLC. Prostate tissues were incubated with PLK inhibitors and equivalent amounts of solvent (DMSO) for 60 min. Two sets of experiments were performed, where effects of SBE 13 (1 μM) and cyclapolin 9 (3 μM), or of Ro 3280 (100 nM) and TAK 960 (100 nM) were assessed. Vimentin phosphorylation at serine 56 and MLC phosphorylation at serine 19 were compared between inhibitor-treated samples and corresponding controls (exposed to identical experimental conditions) by Western blot analysis using phospho-specific antibodies. In each series, tissues in the agonist and corresponding control group were obtained from the same prostates. Shown are representative Western blots and quantification of all experiments, from series with *n* = 8 patients the SBE 13/cyclapolin 9 series, and *n* = 8 other patients for the Ro 3280/TAK960 series (^∗^*p* < 0.05 for control vs. inhibitor).

Incubation of prostate tissues with SBE 13 (1 μM), cyclapolin 9 (3 μM), Ro 3280 (100 nM), or TAK 960 (100 nM) for 60 min reduced the average content of phospho-vimentin (serine 56). This was significant for Ro 3280 (*p* < 0.05) and TAK960 (*p* < 0.009), while a trend was observed for SBE 13 and cyclapolin 9 (**Figure 8**). In contrast, none of the inhibitors changed the content of total vimentin, phospho-MLC (serine 19), or MLC (**Figure 8**).

## Discussion

Our findings suggest that neurogenic and α_1_-adrenergic smooth muscle contraction in the human prostate can be inhibited by PLK inhibitors. In contrast to α_1_-adrenergic contractions, endothelin-1- and thromboxane A_2_-induced contractions were not susceptible to PLK inhibitors, pointing to a divergent regulation of adrenergic and non-adrenergic prostate smooth muscle contraction by PLKs. It appears possible that PLK1 promotes α_1_-adrenergic smooth muscle contractions in the hyperplastic human prostate. This is in line with recent evidence, which suggested that smooth muscle contraction of the human prostate is insufficiently understood. In fact, recent studies reported inhibition of prostate smooth muscle contraction by inhibitors for several kinases or GTPases (including c-Jun N-terminal kinase, focal adhesion kinase, src family kinases, LIM kinases, Rac GTPases) (Strittmatter et al., 2012; Kunit et al., 2014; Wang et al., 2016a,b; Herlemann et al., 2018; Yu et al., 2018), although their involvement of prostate smooth muscle contraction was not known before. This demonstrates that this process is probably more complex than previously assumed, what may be important from a clinical point of view. Considering the insufficient efficacy of available medications for LUTS treatment, the important role of prostate smooth muscle tone for etiology and therapy of male LUTS, together with the high and even increasing relevance of LUTS, proves our understanding of regulation of smooth muscle contraction in the lower urinary tract is in fact highly mandatory to overcome current limitations in future.

Following the discovery of PLK1 as important regulator of mitosis and proliferation in different cell types, PLKs emerged as promising new targets in oncology (McInnes et al., 2005). This initiated the development of PLK-specific small molecule inhibitors, which has been promoted during the last decade and resulted in several inhibitors which are now available for research purposes (Gutteridge et al., 2016). In kinase assays based on immunoprecipitated enzymes, SBE 13 showed high selectivity for PLK1, with an IC_50_ value of 200 pM for PLK1 inhibition, and IC_50_ values of 66 μM and 875 nM for inhibition of PLK2 and -3, while no inhibition of aurora kinase was observed (Keppner et al., 2009, 2010). In different cell lines, including HeLa and cancer cells, proliferation was inhibited with EC_50_ values ranging between 5 and 60 μM (Keppner et al., 2009, 2010). Cyclapolin 9 showed an IC_50_ of 500 nM for PLK1 in *in vitro* kinase assays, while a panel of at least 37 other kinases was not inhibited even at a cyclapolin 9 concentration of 100 μM (McInnes et al., 2006). Ro 3280 inhibited PLK1 with an IC_50_ value of 3 nM in biochemical assays, and 6 nM in H82 cells (Chen et al., 2012). Even at a concentration of 1 μM, Ro 3280 inhibited only 13 out of a panel of 293 tested kinases (Chen et al., 2012). EC_50_ values for the inhibition of proliferation in 13 different tumor cell lines ranged between 6 and 82 nM, with 12 nM for prostatic PC-3 cells (Chen et al., 2012). TAK-960 inhibits PAK1 with an IC_50_ of 1.5–2 nM, and is orally available (Hikichi et al., 2012; Nie et al., 2013). Proliferation of cultured cancer cells was effectively inhibited by TAK-960 in a low, one-digit nanomolar range (Nie et al., 2013). At a concentration of 1 μM, 243 of 288 tested kinases were inhibited by less than 20% (Hikichi et al., 2012). Therefore and based on the concentrations used in our study (1 μM SBE 13; 3 μM cyclapolin 9; 100 nM Ro 3280; 100 nM TAK-960), we assume that the effects we observed in our experiments using human prostate tissues may be attributed largely to inhibition of PLKs, while unspecific kinase inhibition may play a minor role.

In addition to our functional experiments with PLK inhibitors in an organ bath, the presence of active PLK1 in smooth muscle cells of human prostate tissues was suggested by molecular detection. Staining with an antibody for threonine-210-phosphorylated PLK1 resulted in immunoreactivity within stromal smooth muscle cells. Activation of PLK1 requires phosphorylation at this residue, so that this may reflect the presence of active PLK1 (Jang et al., 2002; Paschal et al., 2012). Detection by RT-PCR and Western blot analysis suggested that PLK1 and other isoforms may be present in all analyzed tissues, despite strong variations in expression level. Because a role for smooth muscle contraction may be suspected for PLK1, but not for other isoforms, we confined our Western blot analyses and fluorescence stainings to PLK1 (Li et al., 2016; de Carcer et al., 2017). Our findings may suggest that PLK1 is expressed in prostate tissues from all examined patients, while the expression level varied independently from BPH. This became obvious from correlation analysis including band intensities of assumed PLK1 and PSA bands. PSA was detectable in all samples with varying content, reflecting different degree of BPH in prostates from different patients. Consequently, our tissues may be regarded as hyperplastic, although the degree of BPH may vary. From a clinical point of view, only the hyperplastic state is of relevance, as male LUTS are usually associated with BPH. Previously, numerous studies addressed the role of PLKs for proliferation of prostate cancer cells and for tumor growth in the prostate, while the present study may be the first suggesting expression and a function of PLK1 in non-malignant prostate tissue (Liu et al., 2011; Wissing et al., 2013).

The use of antibodies may be regarded as a possible limitation. In fact, antibodies used in our Western blot analyses and stainings were not validated, so that these results should be considered with care. On the other hand, we only considered bands with sizes matching the expected molecular weight of PLK1. Moreover, our functional and molecular data may confirm each other. Thus, despite several limitations, our data may strongly suggest the presence of PLKs and a role in promotion of smooth muscle regulation in the human prostate, if all of our findings are regarded together. Notably, we observed inhibition of α_1_-adrenergic and EFS-induced contractions by PLK inhibitors despite high variation of PLK1 expression between different prostate tissues, which was suggested by Western blot analyses. This may underline the importance of PLK for regulation of prostate smooth muscle contraction. We used different inhibitors and different agonists, so that the data may confirm each other and that the findings may be valid despite all heterogeneities or despite divergent PLK expression.

Recent studies addressed the role of PLK1 in contraction of airway and vascular smooth muscle. First, contraction of tracheal rings from knock out mice was reduced in organ bath experiments, and reduced airway constriction was observed *in vivo* (Li et al., 2016). More recently, a role of PLK1 for contraction of vascular smooth muscle has been suggested, again using PLK1-deficient mice (de Carcer et al., 2017). *In vivo*, these mice showed hypotension, and reduced angiotensin II-induced hypertension (de Carcer et al., 2017). In organ bath studies, contractile responses to α_1_-agonists and angiotensin II were reduced in the aorta and in mesenteric arteries from PLK1 knock-out mice (de Carcer et al., 2017). Together, these findings prompted us to examine possible effects of PLK inhibitors on prostate smooth muscle contraction.

The role of PLK for promotion of smooth muscle contraction is obviously shared by the prostate, the cardiovascular system and airways (Li et al., 2016; de Carcer et al., 2017). Our findings may point to divergent regulation of PLK activity in different organs, but to shared mechanism underlying this PLK function. Unlike airway smooth muscle, where PLK may be activated by stimulation of cholinergic receptors during acetylcholin-induced contraction (Li et al., 2016), PLK is apparently not activated by contractile receptors in the human prostate. We tested different incubation periods with agonists, considering that kinetics of contraction may differ for α_1_-adrenoceptor agonists and U46619. Although our data suggest active PLK1 in resting prostate smooth muscle, the activating mechanisms remain to be determined and may not be concluded from our present study. On the other hand, intracellular mechanisms underlying PLK-dependent promotion of smooth muscle contraction may be similar in the prostate and airways. Similar to airway smooth muscle, our findings suggest that a mechanism based on vimentin phosphorylation at serine 56 may underly promotion of smooth muscle contraction by PLK in the prostate, while an involvement of myosin light chain (MLC) phosphorylation appears unlikely. In fact, vimentin promotes smooth muscle contraction independently from MLC phosphorylation (Wang et al., 2006; Li et al., 2016). Although our findings addressing intracellular mechanisms of PLK actions in prostate smooth muscle contraction are to some extent still preliminary, this may provide a basis for further investigations, including the role of vimentin in prostate smooth muscle cells.

Worldwide, 600 million patients with LUTS suggestive of BPH are expected in 2018, paralleled by expenses of probably more than 4.7 billion USD for medical treatment of male LUTS (Irwin et al., 2011; Ventura et al., 2011). Case numbers, expenses, and relevance of LUTS will even further increase, because the prevalence of LUTS increases with age and due to the demographic transition at least in Western countries. This is contrasted by the limited efficacy of available drugs to improve urinary flow (Q_max_) or international prostate symptom scores (IPSS) by not more than 50% (Oelke et al., 2013; Hennenberg et al., 2014, 2017). In fact, disappointing results of medical LUTS therapy contributes to exceedingly high discontinuation rates, peaking up to 70% of patients discontinuing their medication within 12 month following first prescription (Cindolo et al., 2015). This may result in disease progression, hospitalization, and surgery for BPH (Cindolo et al., 2015). Considering all this together with the role of prostate smooth muscle contraction for pathophysiology and therapy of male LUTS, improved understanding of prostate smooth muscle contraction becomes increasingly important.

Our current findings are in line with recent studies suggesting that the contractile mechanisms of prostate smooth muscle are incompletely understood. For more than one decade, it has been assumed that prostate smooth muscle contraction is promoted by three intracellular signaling pathways, including inositol-1,4,5-trisphosphate/calcium, diacylglycerol/protein kinase C, and RhoA/Rho kinase (Andersson et al., 1997; Christ and Andersson, 2007). It has now become clear, that mechanisms of prostate smooth muscle contraction are much more complex and encompass further pathways and mediators, including several kinases, GTPases, and their activators (Strittmatter et al., 2011; Kunit et al., 2014; Wang et al., 2015, 2016a,b). Our current findings suggest that the network of procontractile signaling pathways in the prostate includes PLKs. This ongoing, recent discovery of new contraction mechanisms in the prostate is paralleled by research in other organs, being characterized by a similar continuous description of new mediators of smooth muscle contraction.

Non-adrenergic mediators, particularly endothelin-1 and thromboxane A_2_, may contribute to prostate smooth muscle contraction in parallel to α_1_-adrenoceptors. It is assumed that their contributions to prostate smooth muscle tone are responsible for the limitations of α_1_-blockers, as they will not inhibit endothelin- or thromboxane-induced contractions and may improve LUTS by maximally 50% (Hennenberg et al., 2013, 2014, 2017). The importance of these non-adrenergic mediators for prostate smooth muscle tone has recently become clear (Hennenberg et al., 2013, 2014, 2017; Strittmatter et al., 2011). Due to the assumed high relevance of non-adrenergic mediators for etiology and therapy of LUTS suggestive of BPH, adequate understanding of their contractile mechanisms may be very much appreciated. However, compared to α_1_-adrenergic prostate contraction, they are less understood and only little is known about the underlying mechanisms. Certainly, adrenergic and non-adrenergic prostate contraction share some common intracellular pathways, e.g., signaling by calcium and Rho kinase, which are used by α_1_-adrenoceptors, as well as thromboxane and endothelin receptors to induce smooth muscle contraction in the prostate (Christ and Andersson, 2007; Takahashi et al., 2007; Strittmatter et al., 2011). However, our present study demonstrates that adrenergic and non-adrenergic contractions may be differentially promoted and regulated, apart from shared intracellular mechanisms of receptor-induced contraction. Obviously, a PLK inhibitor-sensitive mechanism confers differential regulation of α_1_-adrenergic and non-adrenergic prostate contraction in the human prostate. Understanding such differences is mandatory to grasp regulation and mechanisms of non-adrenergic contraction in the context of LUTS treatment, and to address it in future therapies.

## Conclusion

Alpha_1_-adrenergic smooth muscle contraction in the human prostate can be inhibited by PLK inhibitors. PLK-dependent signaling may be a new pathway, which promotes contraction of prostate smooth muscle cells. As contractions by endothelin and thromboxane A_2_ are not susceptible to PLK inhibition, this reflects differences in promotion of adrenergic and non-adrenergic prostate smooth muscle contraction.

## Author Contributions

All authors contributed to experiments and to acquisition of data. MH, PK, QY, YW, BR, and AC contributed analysis and interpretation of data. MH, QY, AH, and AT contributed to drafting of the manuscript. MH, FS, CS, and CG contributed to conception of the work. YW, FS, CS, and CG critically revised the manuscript for important intellectual content. All authors approved the final version of the manuscript, and provided agreement to be accountable for all aspects of the work in ensuring that questions related to the accuracy or integrity of any part of the work are appropriately investigated and resolved.

## Conflict of Interest Statement

The authors declare that the research was conducted in the absence of any commercial or financial relationships that could be construed as a potential conflict of interest.
